# Phosphorylation of IRS1 at Serine 307 in Response to Insulin in Human Adipocytes Is Not Likely to be Catalyzed by p70 Ribosomal S6 Kinase

**DOI:** 10.1371/journal.pone.0059725

**Published:** 2013-04-02

**Authors:** Meenu Rohini Rajan, Siri Fagerholm, Cecilia Jönsson, Preben Kjølhede, Maria V. Turkina, Peter Strålfors

**Affiliations:** Department of Clinical and Experimental Medicine, Linköping University, Linköping, Sweden; Tohoku University, Japan

## Abstract

The insulin receptor substrate-1 (IRS1) is phosphorylated on serine 307 (human sequence, corresponding to murine serine 302) in response to insulin as part of a feedback loop that controls IRS1 phosphorylation on tyrosine residues by the insulin receptor. This in turn directly affects downstream signaling and is in human adipocytes implicated in the pathogenesis of insulin resistance and type 2 diabetes. The phosphorylation is inhibited by rapamycin, a specific inhibitor of mammalian target of rapamycin (mTOR) in complex with raptor (mTORC1). The mTORC1-downstream p70 ribosomal protein S6 kinase (S6K1), which is activated by insulin, can phosphorylate IRS1 at serine 307 in vitro and is considered the physiological protein kinase. Because the IRS1 serine 307-kinase catalyzes a critical step in the control of insulin signaling and constitutes a potential target for treatment of insulin resistance, it is important to know whether S6K1 is the physiological serine 307-kinase or not. We report that, by several criteria, S6K1 does not phosphorylate IRS1 at serine 307 in response to insulin in intact human primary adipocytes: (i) The time-courses for phosphorylation of S6K1 and its phosphorylation of S6 are not compatible with the phosphorylation of IRS1 at serine 307; (ii) A dominant-negative construct of S6K1 inhibits the phosphorylation of S6, without effect on the phosphorylation of IRS1 at serine 307; (iii) The specific inhibitor of S6K1 PF-4708671 inhibits the phosphorylation of S6, without effect on phosphorylation of IRS1 at serine 307. mTOR-immunoprecipitates from insulin-stimulated adipocytes contains an unidentified protein kinase specific for phosphorylation of IRS1 at serine 307, but it is not mTOR or S6K1.

## Introduction

As part of insulin signaling, the insulin receptor substrate-1 (IRS1) is phosphorylated directly by the insulin receptor on specific tyrosine residues. IRS1 serves as a hub for transmitting the insulin signal, for example, to metabolic control of the cell via protein kinase B (PKB) and mitogenic control via MAP-kinases ERK1/2. IRS1 is also important as a target for modification of the insulin signal through crosstalk with other signaling pathways and through downstream feedback signals in response to insulin. Many of these modifying signals impinge on IRS1 through phosphorylation of serine and threonine residues, reviewed in [1]–[5].

Much of the phosphorylation of IRS1 at serine/threonine residues has been associated with inhibition of insulin signaling, either through interference with insulin receptor phosphorylation of IRS1 at tyrosine, binding of downstream signaling intermediaries and/or through increased IRS1 degradation [6]–[22]. But, phosphorylation of IRS1 at serine/threonine has also been shown to enhance the tyrosine phosphorylation of IRS1 in response to insulin and hence to stimulate insulin signaling [23]–[32]. Phosphorylation of IRS1 at serine 307 (Ser307, human sequence, corresponding to serine 302 in the murine sequence) has been extensively examined, in particular as part of a feedback loop in insulin signaling but also as input from other signaling pathways. Phosphorylation of IRS1 at Ser307 is of interest as it has been implicated in a causative mechanism of insulin resistance, as part of negative [22], [33]–[36] or positive control signals [27]–[30], [37]. While phosphorylation at Ser307 in the control of insulin signaling is undisputed, whether it is part of a positive [27]–[30], [37] or negative [36] feedback is not agreed. There are indications that phosphorylation at Ser307 blocks phosphotyrosine protein phosphatases from dephosphorylating and inactivating IRS1 [30]. The importance of cell type and experimental conditions associated with attributing positive or negative effects of a feedback to phosphorylation of IRS1 is illustrated in the case of phosphorylation of IRS1 at serine 312 (serine 307 in murine sequence) – an established negative feedback in different experimental set ups [4], [6], [12] that in a knock-in experiment eventually was found to have a positive effect on IRS1 and insulin signaling in mice *in vivo* [31].

Ser307 is found in a consensus sequence for phosphorylation by PKB or the p70 ribosomal S6 kinase (S6K1). Insulin induced phosphorylation at Ser307 is acutely inhibited by rapamycin [28], [29], a specific inhibitor of mammalian target of rapamycin (mTOR) in complex with raptor (mTORC1), which argues against protein kinase B as the physiologically relevant insulin-stimulated Ser307-kinase. S6K1 was found to phosphorylate a large fragment of IRS1 at Ser307 in vitro, and insulin-induced phosphorylation was blocked by knock-down of S6K1 in TSC2^−/−^ mouse embryo fibroblasts [36]. As S6K1 is phosphorylated and activated by mTORC1, S6K1 is widely regarded as the physiological Ser307-kinase. We set out to examine this proposition in primary human adipocytes, which are primary target cells of insulin and where attenuated phosphorylation of Ser307 is critical in the pathogenesis of insulin resistance and type 2 diabetes [28], [37]. Such a role of phosphorylation of IRS1 at Ser307 makes the Ser307-kinase a potential target for treatment of insulin resistance. We now report that by several criteria S6K1 is unlikely to be the protein kinase that phosphorylates IRS1 at Ser307 in response to insulin in mature human primary adipocytes.

## Materials and Methods

### Ethics Statement

The study was approved by the Regional Ethics Board at Linköping University; all patients obtained written information and gave their informed verbal approval before the surgery. Verbal approval was considered sufficient by the ethics board considering that a small piece of adipose tissue was obtained during elective surgery for other reasons, and that the procedure posed no discomfort or threat to the health of the patients. The procedure was documented as part of the surgical protocol and adipose tissues samples were anonymized.

### Materials

Antibodies against IRS1 phospho-Ser307 (#2384S), S6 ribosomal protein phospho-Ser235/236 (#2211S), S6 ribosomal protein (5G10, #2217), p70S6K (#9202), and mTOR (#2972) were from Cell Signaling Technology (Danvers, MA, USA). Anti-beta-Tubulin (#T4026) was obtained from Sigma Aldrich (St. Louis, MO, USA), anti-IRS1 (06–248) from Millipore (Schwalbach, Germany) and anti-Raptor (A300–506A) from Bethyl Laboratories, Inc. (Montgomery, TX, USA). PCR primers and restriction enzymes were obtained from Invitrogen Life Science (Scotland, UK). QuikChange Site-Directed Mutagenesis Kit was from Stratagene. PF-4708671 and the rest of the chemicals were from Sigma Aldrich unless otherwise indicated in the text.

### Isolation of Human Adipocytes

Subcutaneous adipose tissue was obtained during elective abdominal surgery from patients under general anesthesia. Patients were not diagnosed with diabetes and were within the body mass index range of 18–28 Kg/m^2^.

Adipocytes were isolated by collagenase digestion (Type I, Worthington, NJ, USA) as described in [38] and washed in supplemented Krebs Ringer solution as described in [39]. The cells were incubated overnight in the same solution mixed with DMEM containing 7% albumin, 200 nM phenylisopropyladenosine, 20 mM Hepes, 50 UI/ml penicillin, 50 µg/ml streptomycin, pH 7.40, at 37°C. Before final analysis, cells were washed and preincubated in the Krebs Ringer solution supplemented with final concentration of 100 nM phenylisopropyladenosine, 0.5 U**·**mL^−1^ adenosine deaminase for 20 min, before incubation with additions as indicated.

### Generation of Recombinant Adenovirus Carrying Dominant Negative S6K1

Adenovirus expression plasmid pAD/CMV/V5-DEST carrying dominant negative S6K1 or S6K1-DN (T389A) was a generous gift from Jianping Ye, Pennington Biomedical Research Center, Louisiana. pAd/CMV/V5-DEST EGFP was from Invitrogen (Carlsbad, CA, USA). Adenoviruses were amplified, harvested and titrated according to ViraPower Adenoviral Expression System protocol, Invitrogen.

### Transfection of Adipocytes

For transfection, the isolated adipocytes were incubated in DMEM supplemented with 1% BSA, 1% Penicillin-Streptomycin, 200 nM adenosine, 7.5 mM glucose and 10% fetal calf serum (Invitrogen) for 1 h at 37°C. Adipocytes were then transduced with S6K1-DN adenovirus (MOI 30) for 48 h with serum starvation for 18 h prior to incubation with10 nM insulin for indicated time periods. Untransfected and EGFP adenovirus infected cells were used as controls.

### Immunoprecipitation

Adipocytes were lysed in 2 vol lysis buffer (25 mM Tris-Cl, pH 7.4, 100 mM NaCl, 1 mM EGTA, 0.1% Tween-20, 10 µM Leupeptin, 1 µM Pepstatin, 1 µM Aprotinin, 50 µM phenylmethanesulfonyl fluoride) by passing three times through a 27 gauge needle. The lysate was immediately put on ice, and cell debris and fat was removed after centrifugation for 10 min at 500×g at 4°C. The infranatant was additionally centrifuged for 30 min at 20000×g, 4°C to remove the last traces of fat and then stored at −80°C. Lysate with 0.2 mg protein was pre-cleared on 25 µL Protein G PLUS-Agarose (sc-2002, Santa Cruz Biotechnology, Santa Cruz, CA, USA) for 30 min of rotation end-over-end at 4°C and then incubated overnight with 4 µL anti-mTOR antibody rotating end-over-end at 4°C. 25 µL Protein G PLUS-Agarose was then added to the anti-mTOR-incubated lysates and incubated for 4 h, rotating end-over-end at 4°C. Precipitates were washed three times with lysis buffer and then prepared for SDS-PAGE or for S307-kinase assay.

### SDS-PAGE and Immunoblotting

**Figure 1:**
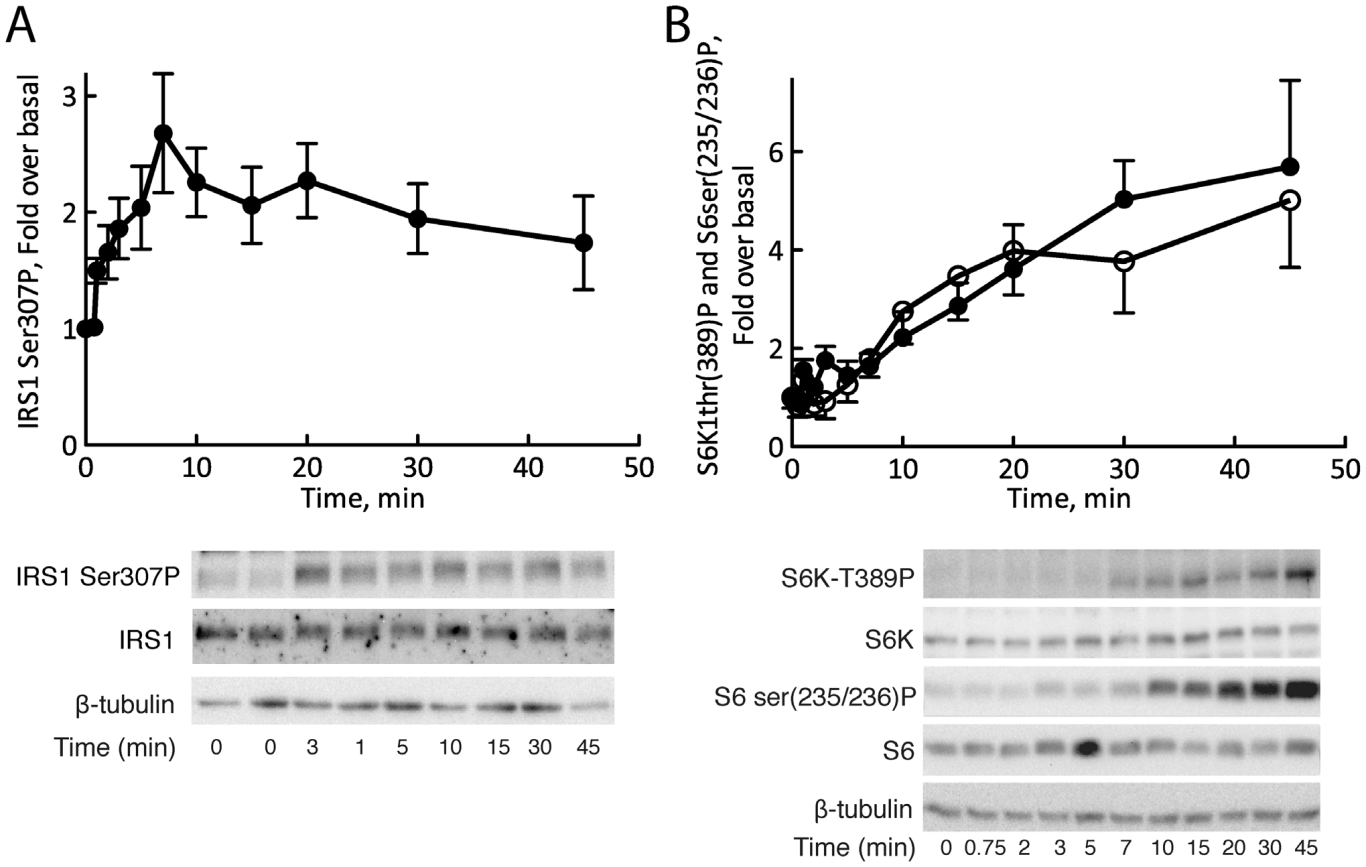
Time-course for insulin induced phosphorylation of S6, S6K1, and of IRS1 at Ser307. Isolated human primary adipocytes were stimulated with 10 nM insulin for the indicated time periods and then resolved by SDS-PAGE followed by immunoblotting with antibodies against (A) IRS1 phospho-Ser307 and (B) S6 phospho-Ser235/236 (filled circles) and S6K1 phospho-Thr389 (open circles). Quantified phosphorylation data are expressed as fold over basal without insulin. Mean±SE, n = 6 subjects (A) and n = 5 subjects (B). Also shown are representative immunoblots.

Cell incubations were terminated by separating cells from medium by centrifugation through diisononylphtalate. To minimize post-incubation modifications of proteins, the cells were immediately dissolved in SDS and β-mercaptoethanol with protease and protein phosphatase inhibitors, and rapidly frozen. Cell samples were thawed by boiling, and immunoprecipitates boiled, in SDS-PAGE sample buffer for 3 min and resolved by SDS-PAGE. Proteins were transferred to PVDF membranes (Immobilon-P, Millipore) and immunoblotted with indicated antibodies. The signal was detected by chemiluminiscence (ECL+, GE Healthcare) imaging (Las 1000, Fujifilm, Tokyo, Japan) and analyzed using imaging software MultiGauge V3.0 (Fujifilm, Tokyo, Japan). In this study we examine acute effects of insulin on protein phosphorylation, and since the total amount of IRS1, S6K or S6 protein doesńt change (Fig. 1) we chose to normalize for the amount of β-tubulin to control for any variation in the amount of specific protein loaded. Statistical analysis was performed using GraphPad Prism5 (La Jolla, CA, USA). Statistical difference between two groups was analyzed using two-tailed Student’s paired t-test and P<0.05 was considered to be statistically significant.

### Production of S307-peptide and A307-peptide

Human IRS-1 S307A mutant was obtained by PCR and QuikChange Site-Directed Mutagenesis Kit from the plasmid pSIS2-hIRS-1-HA using the following primers: 5′-TCACGCACTGAAGCCATCACCGCCACCTCC and 5′-CGATCACGCACTGAAGACATCACCGCCACCTCC, respectively (altered bases are underlined). These primers also created an *Eco* 571 restriction site for identification. Positive clones were verified by sequencing. In order to create plasmids encoding GST-fusion proteins, cDNA with these mutations or the wild-type sequence, encoding aa 288–314 was amplified by PCR using the following primers: 5′-TGCGCCCGGGCCATCTCAACAATC and 5′-CCCAGAATTCTGGCCGGGGAGGT, cut with *Xma* I and *Eco* RI and subcloned into the pGEX-JD plasmid.

The vectors were transformed into competent *E. coli* Y1090 and GST-fusion protein production was induced with 1 mM isopropyl β-thiogalactoside. Cells were lysed through sonication and lysate was cleared (centrifugation at 25000 rpm, 30 min, 4°C). Lysate was diluted in NET-N buffer (50 mM Tris-Cl, pH 7.4, 150 mM NaCl, 5 mM EDTA and 0.5% NP-40) and GST-peptides were captured on GSH-sepharose beads for 30 min at 4°C. The beads were washed twice in NET-N buffer and once in 50 mM NH_4_HCO_3_ with 2 mM CaCl_2_. The peptides were cleaved off the GST-tag with 1 unit thrombin (Amersham Pharmacia Biotech Inc., Piscataway, NJ, USA) in a total volume of 1 ml for 2 h at 22°C.

### Ser307-kinase Assay

Immunoprecipitates of mTOR were incubated with the S307-peptide and 50 µM ATP in the presence of 10 mM MgCl_2_, 50 mM KCl and 25 mM Hepes at pH 7.4 for 2 h at 30°C, with or without 0.5 µM Torin (Tocris Bioscience, Bristol, UK), 20 µM PF-4708671 (PZ0143, Sigma Chem. Co), or 3 µM Akti1/2 (sc-202048 Santa Cruz Biotechnology, Santa Cruz, CA, USA), as indicated. Incubations were terminated by isolating the peptides on a C18 ZipTip (Millipore, Schwalbach, Germany) followed by digestion with trypsin (90055, Thermo scientific, Rockford, IL, USA) for 2 h at 37°C. Trypsinated peptides were dried and resolved in 0.1% Formic Acid before being subjected to LC-MS/MS analysis.

### Peptide Analyses by LC-MS/MS

The obtained peptide mixtures were analyzed by LC-MS/MS. Separation was done using nano-flow HPLC system (EASY-nLC, Bruker Daltonics, Bremen, Germany) and data were acquired using on-line electrospray ionization ion trap“HCTultra PTM Discovery System” (Bruker Daltonics). Peptides were separated during 45 min by reverse phase chromatography on a 20 mm×100 µm C18 pre column followed by a 100 mm×75 µm C18 column (particle size 5 µm, NanoSeparations, Nieuwkoop, Netherlands) at a flow rate 300 nL/min. A gradient of 0.1% formic acid in water (A) and 0.1% formic acid in acetonitrile (B) was distributed as follows: starting with 4% B; linear gradient 4%–20% B in 0–25 min; 20%–100% B in 25–35 min and 100% B in 35–45 min.

Automated online tandem MS analyses were performed using alternating collision induced dissociation (CID) and electron transfer dissociation (ETD) of peptide ions. Peak lists were created from the raw data using DataAnalysis 3.4 (Bruker Daltonics) and the resulting MGF files were used to search for *Homo sapiens* proteins in Swissprot database on a public Mascot server (www.matrixscience.com) with a second-pass Mascot error tolerant search to detect protein sequence variants with amino acid substitutions. The search parameters allowed mass errors up to 0.6 Da for MS data, and up to 0.6 Da for MS/MS data in case of CID of peptide ions and 1.5 Da in case of ETD of peptide ions. The charge states of the peptides were varied; and three missed cleavage sites were permitted. Serine and threonine phosphorylation were selected as variable modifications. The sequence of each peptide was verified by manual analysis of MS/MS spectra.

### Relative Quantification of S307-peptide Phosphorylation Using Mass Spectrometry

**Figure 2:**
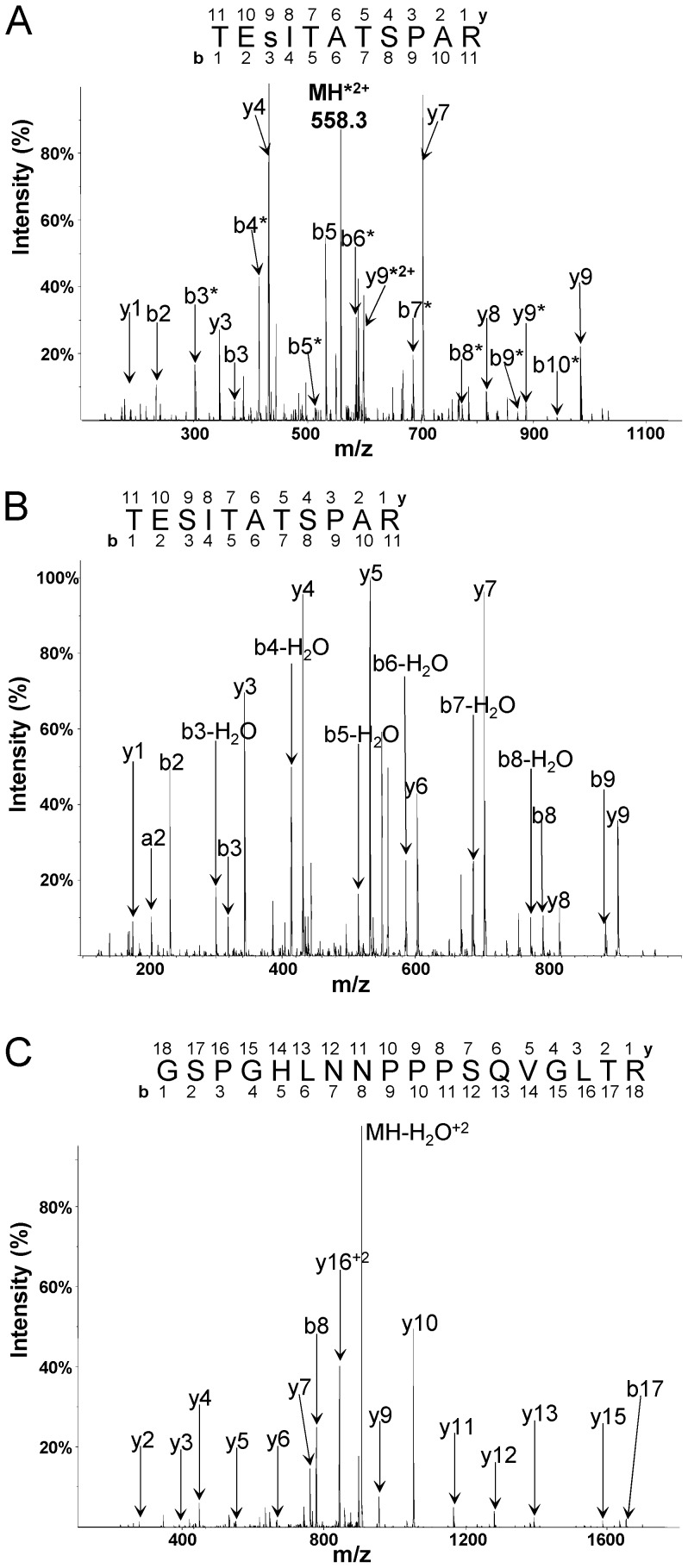
Characterization of the phosphorylation site corresponding to Ser307 of IRS1 in S307-peptide by mass spectrometric sequencing. (A) CID spectrum of doubly charged phosphopeptide TEsITATSPAR (originated from S307-peptide phosphorylated by mTOR-immunoprecipitate in phosphorylation assay) with M/Z 607.3. The doubly charged ion indicated at M/Z = 558.3 corresponds to the parent ion after the neutral loss of phosphoric acid (H_3_PO_4_; 98 Da). The b (N-terminal) and y (C-terminal) fragment ions are labeled in the spectrum and in the displayed peptide sequence. Lowercase s in the sequence designates phosphorylated serine residue. Phosphorylation site was localized according to the pattern of the fragment ions containing phosphate and corresponding fragments with the neutral loss, which are marked with asterisks. Phosphorylation of the serine residue is evident from the distinct set of the fragments: b3, b3*, b4*, y9, y9*and y9*^2+^. (B) CID spectrum of doubly charged peptide TESITATSPAR (originated from S307-peptide) with mass over charge ratio (M/Z) 567.3. The b (N-terminal) and y (C-terminal) fragment ions are labeled in the spectrum and in the displayed peptide sequence. (C) CID spectrum of doubly charged peptide GSPGHLNNPPPSQVGLTR with M/Z 914.5. The b (N-terminal) and y (C-terminal) fragment ions are labeled in the spectrum and in the displayed peptide sequence.

For the comparative quantification of phosphorylation of the S307-peptide, peptide mixtures from the Ser307-kinase assay were analyzed directly by LC-MS/MS using 45 min LC-method. The extracted ion chromatograms acquired were computed for the selected peptide ions. Ion chromatograms of tryptic peptides originated from S307-peptide TESITATSPAR, with M/Z 567.3, (Fig. 2B) and corresponding phosphorylated peptide TEsITATSPAR, with M/Z 607.3, (Fig. 2A) were monitored in parallel. Tryptic peptide GSPGHLNNPPPSQVGLTR, with M/Z 914.5, (Fig. 2C) from S307-peptide was also monitored as an internal standard in each LC-run to normalize for the amount of peptide loaded.

The ion chromatogram peak area represents the total ion abundance of the selected peptide. Analyses and determination of areas of each individual extracted ion chromatogram were performed using DataAnalysis 3.4 (Bruker Daltonics, Bremen, Germany). The resulting data are expressed as a percentage relative control incubations with no additions (vehicle). Statistical difference between a treatment and the control group was analyzed using one-sample t-test and P<0.05 was considered to be statistically significant.

## Results and Discussion

### S6K is not Phosphorylating IRS1 at Ser307 in Intact Adipocytes

We examined human primary mature adipocytes, which represent a primary cell type for insulin action and the development of insulin resistance in obesity. We first analyzed the time-course for phosphorylation of IRS1 at Ser307 in response to a maximal effective concentration of insulin, and compared it with that of the phosphorylation of the ribosomal protein S6 (S6) at serine 235/236, which is a bona fide physiological substrate of S6K1. The phosphorylation of IRS1 at Ser307 was rapid, reaching a quasi steady-state extent of phosphorylation after 5 min (Fig. 1A). In contrast, the phosphorylation of S6 at serine 235/236 exhibited a distinct lag-time that lasted for 5 min, after which the phosphorylation slowly increased to reach a quasi steady-state level after 30 min (Fig. 1B). To examine if the low rate of phosphorylation of S6 was limited by the upstream phosphorylation and activation of S6K1, we determined also the time-course for phosphorylation of S6K1 at threonine 389 (Fig. 1B). The phosphorylation of S6 closely reflected the phosphorylation and hence activation state of S6K1, which thus was also much slower than that of IRS1 at Ser307. The slow dynamics for phosphorylation/activation of S6K1 is not unique to adipocytes as it has been described also in, e.g., Hela cells [40]. This would preclude differences in the rates of dephosphorylation of S6-P and IRS1-Ser307-P as an explanation of the different time-courses. The very different dynamics for phosphorylation of IRS1 at Ser307, on one hand, and of S6K1 at threonine 389 and S6 at serine 235/236, on the other, suggest that IRS1 is not phosphorylated at Ser307 by S6K1. It is, however, conceivable that IRS1-Ser307 is a much better, or co-localized, substrate of S6K1 than S6, such that very little S6K1 needs be phosphorylated/activated to affect the phosphorylation of IRS1 at Ser307, while essentially no S6 is phosphorylated. We therefore next examined the effect of expressing a dominant-negative mutant of S6K1 on the phosphorylation of S6 and IRS1-Ser307 in the human adipocytes.

**Figure 3:**
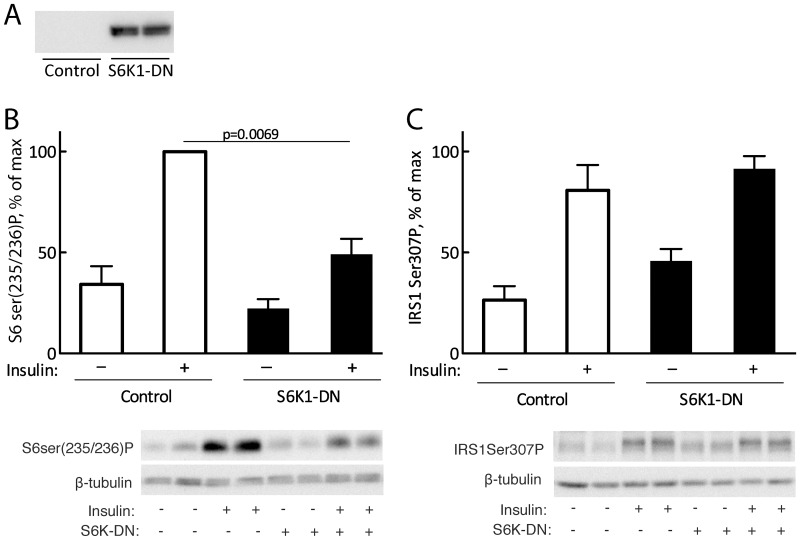
Effect of S6K1-DN mutant on phosphorylation of S6 and of IRS1 at Ser307. (A) Overexpression of S6K1 total protein. Isolated human primary adipocytes were infected with S6K1-DN adenovirus. Untransfected cells were used as control. Cell lysates were subjected to SDS-PAGE and immunoblotting with antibodies against S6K1. The blot is representative of the four independent experiments in (B,C). (B,C) Isolated human primary adipocytes were infected with S6K1-DN adenovirus. Untransfected cells were used as control. Cells were incubated with 10 nM insulin for 40 min (B) or 10 min (C). Whole cell lysates were subjected to SDS-PAGE and immunoblotted with antibodies against S6 phospho-Ser235/236 (B) or IRS1 phospho-Ser307 (C). Mean±SE (n = 4). Also shown are representative immunoblots.

Cells were transfected with a dominant negative construct of S6K1 (S6K1-DN) with the mTORC1 phosphorylation site at threonine 389 substituted with alanine, which precludes activation of the S6K1-DN by insulin. Overexpression of S6K1-DN (Fig. 3A) inhibited the insulin stimulated increase in phosphorylation of S6 at serine 235/236 (Fig. 3B), without effect on the insulin-induced phosphorylation of IRS1 at Ser307 (Fig. 3C). Transfection with the adenovirus vector carrying EGFP had no effect on the insulin-stimulated phosphorylation of either S6 or of IRS1-Ser307 (Fig. S1).

**Figure 4:**
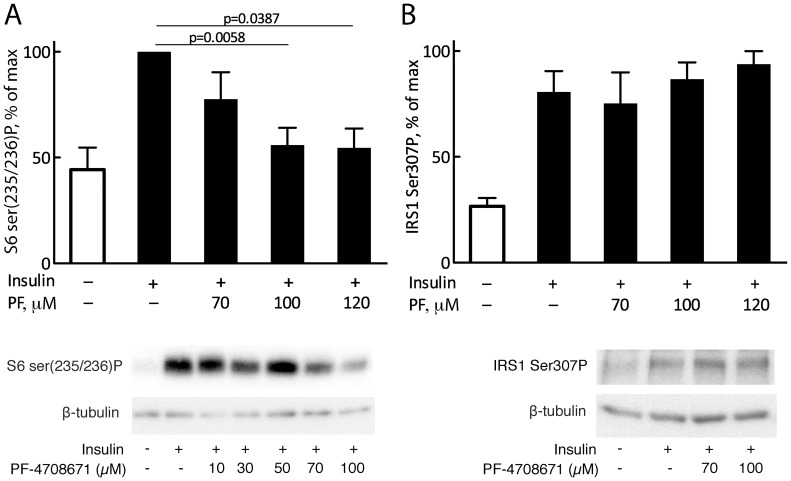
Effect of S6K1 inhibitor PF-4708671 on insulin induced phosphorylation of S6 and of IRS1 at Ser307. Isolated human primary adipocytes were preincubated with indicated concentrations of PF-4708671 (PF) for 30 min and then incubated with 10 nM insulin for 40 min (A) or 10 min (B). Whole cell lysates were subjected to SDS-PAGE and immunoblotting against S6 phospho-Ser235/236 (A) or IRS1 phospho-Ser307 (B). Mean±SE (n = 4). Also shown are representative immunoblots.

As it is possible that the S6K1-DN may have different binding characteristics for different substrates, we next examined also the effect of the S6K1 specific inhibitor PF-4708671 [41]. The inhibitor in a dose-dependent manner inhibited the insulin-induced phosphorylation of S6 at serine 235/236 (Fig. 4A), but had no effect on the insulin-induced phosphorylation of IRS1 at Ser307 (Fig. 4B). The high concentrations of PF-4708671 that we used should affect also the minor isoform S6K2 but not with complete inhibition [41]. Although S6K2 has predominantly nuclear localization [39], we therefore cannot rule out that S6K2 phosphorylates IRS1 in response to insulin.

The non-compatible time-courses, the selective effect of S6K1-DN, and the selective effect of the S6K1 inhibitor, each analyzes a very different aspect of signaling by S6K1 and each indicates non-compatibility with S6K1 phosphorylating IRS1 at Ser307. Taken together these findings therefore demonstrate that S6K1 is unlikely to be the physiological protein kinase for phosphorylation of IRS1 at Ser307 in response to insulin in human primary adipocytes.

### mTOR-immunoprecipitate Contains a Ser307-kinase, but it is not mTOR

**Figure 5:**
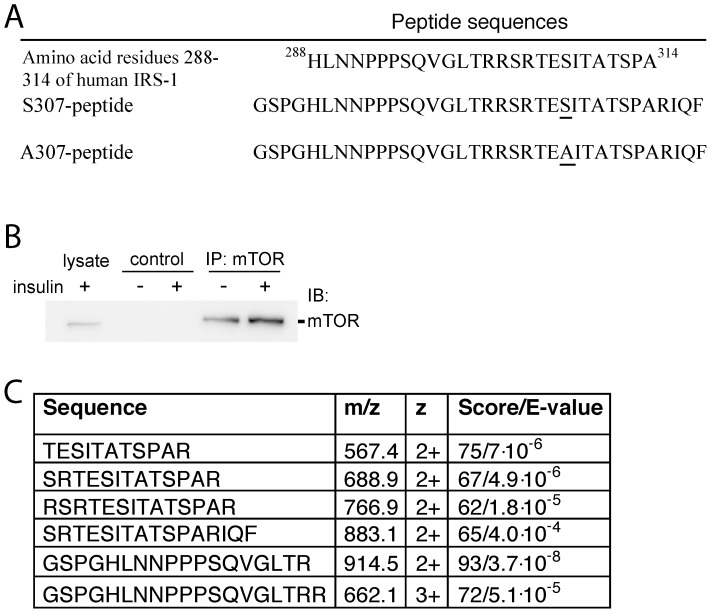
Phosphorylation of S307-peptide by mTOR-immunoprecipitate. (A) Amino acid residues 288–314 of human IRS1 and sequence of S307- and A307-peptides. (B) mTOR immunoprecipitation. Human primary adipocytes were stimulated with insulin for 30 min before cell lysis (two separate immunoprecipitates from the same lysate are shown). Lysates were immunoprecipitated with anti-mTOR antibody. The immunoprecipitate and lysate were analyzed by immunoblotting with anti-mTOR. As a negative control lysate was incubated with Protein G PLUS-Agarose beads without primary antibody. (C) Peptides obtained from limited tryptic proteolysis of the S307-peptide, after incubation with mTOR-immunoprecipitate in phosphorylation assay, identified by LC-MS/MS.

Inhibition of mTORC1 with rapamycin, acutely blocks insulin stimulation of the phosphorylation of Ser307 in intact cells [28], [29], we therefore examined the possibility that mTOR directly phosphorylates Ser307 in an *in vitro* assay. We produced a peptide (S307-peptide) that contains amino acid residues 288–314 of human IRS1 (Fig. 5A). We then immunoprecipitated mTOR from lysates of insulin-stimulated human adipocytes (Fig. 5B) and incubated it with the S307-peptide in the presence of ATP-Mg. After limited proteolysis the obtained peptides were analyzed by LC-MS/MS. We detected and sequenced 6 overlapping peptides, with missed cleavages, which covered the whole S307-peptide sequence (Fig. 5C). The S307-peptide contained 5 serine and 4 threonine residues, but only the serine residue corresponding to Ser307 was phosphorylated by the mTOR-immunoprecipitate (Fig. 2A). As a control, the experiment was repeated with a mutant peptide (A307-peptide) with alanine in place of serine at the position 307 (Fig. 5A). In this case no phosphorylation of the peptide was detected (not shown). This indicates that the mTOR-immunoprecipitate is specific for phosphorylation of Ser307. For the further quantitative analyses of Ser307 phosphorylation we selected the tryptic peptide TEsITATSPAR (lower case denotes phosphorylated residue, Fig. 2A), obtained after 2 h tryptic cleavage to avoid missed cleavages.

**Figure 6:**
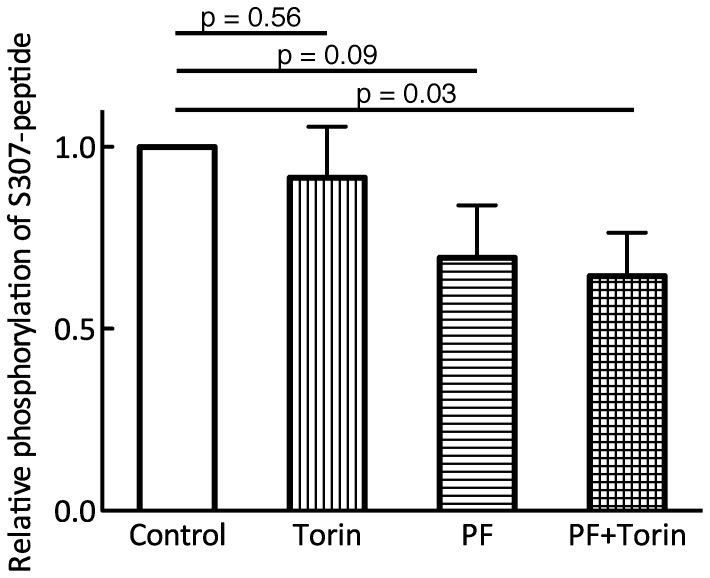
Inhibition of mTOR kinase activity in vitro does not inhibit S307-peptide phosphorylation. Human adipocytes were stimulated with 10 nM insulin for 30 min before cell lysis. Lysate was immunoprecipitated with anti-mTOR antibody. Immunoprecipitates were subjected to a Ser307-kinase assay using the S307-peptide as substrate, in the presence of 0.5 µM torin and/or 20 µM PF-4708671 (PF) for 2 h, as indicated. Phosphorylation of S307-peptide was quantified by LC-MS/MS. Each sample was run in duplicate and their averages were used to calculate the mean±SE (n = 9).

To examine if mTOR was the responsible kinase we included Torin, an ATP-competing inhibitor of mTOR. The extent of phosphorylation of Ser307 by LC-MS/MS was quantified relative the NH_2_-terminal tryptic peptide of the S307-peptide. Although we incubated with a high concentration of Torin (0.5 µM) and a low concentration of ATP (50 µM), phosphorylation of the S307-peptide was not consistently inhibited (Fig. 6). This indicated that mTOR (mTORC1 or mTORC2) in the immunoprecipitate is not the Ser307-kinase. This does not preclude that the phosphorylation is catalyzed by an mTOR-activated protein kinase, as cells were preincubated with insulin to activate mTOR prior to isolation of mTOR and associated protein kinase by immunoprecipitation. Inclusion of the S6K1 inhibitor PF-4708671 appeared to have a small, but not significant, inhibitory effect, indicating the presence of S6K1 in the mTOR-immunoprecipitate. Also in the presence of PF-4708671 Torin had no effect (Fig. 6). A maximum inhibition of 30% in the presence of both inhibitors indicates the presence of an unidentified Ser307-kinase in the mTOR-immunoprecipitate. As PKB also is a potential S307-kinase we repeated the S307-peptide phosphorylation assay in the presence of the PKB inhibitor Akti1/2 at 3 µM, but with no effect on the phosphorylation (not shown).

Our findings demonstrate that it is unlikely that S6K1 is the protein kinase that phosphorylates IRS1 at Ser307 in response to insulin stimulation of human adipocytes. This is interesting as Ser307 presents a consensus recognition site for S6K1 and S6K1 has been shown to phosphorylate an IRS1 fragment (residues 108–516) at Ser307 in an in vitro-assay [36]. The S6K1 is obviously active in the insulin-stimulated adipocytes. The reason Ser307 is not phosphorylated in intact cells may be compartmentalization of the protein kinase and its potential substrate in non-communicating compartments or interference from other proteins. JNK [33] and PKCδ [42] have also been suggested as possible Ser307-kinases, although JNK was refuted as a Ser307-kinase [29], but neither is known to act downstream mTORC1. The specific Ser307-kinase activity of the mTOR-immunoprecipitate is a candidate for the Ser307-kinase. It remains, however, to identify the protein kinase and to examine if it phosphorylates IRS1 at Ser307 in intact human adipocytes.

## Supporting Information

Figure S1 **Overexpression of adenovirus expressing EGFP does not affect S6 or IRS1 Ser307 phosphorylation.** Isolated human primary adipocytes were infected with EGFP adenovirus. Untransfected cells were used as control. Cells were stimulated with 10 nM insulin for 40 min (A) or 10 min (B). Whole cell lysates were subjected to SDS-PAGE and immunoblotted against S6 phospho-ser235/236 (A) and IRS1 phospho-ser307 (B). Mean±SE (n = 3).
(PDF)
