# Supplementary figures and images for: Phosphorylation of IRS1 at Serine 307 in Response to Insulin in Human Adipocytes Is Not Likely to be Catalyzed by p70 Ribosomal S6 Kinase
Source: PLoS One. 2013 Apr 2;8(4):e59725. doi: 10.1371/journal.pone.0059725 (PMC3614923; doi:10.1371/journal.pone.0059725)

# Supporting information

Figure S1

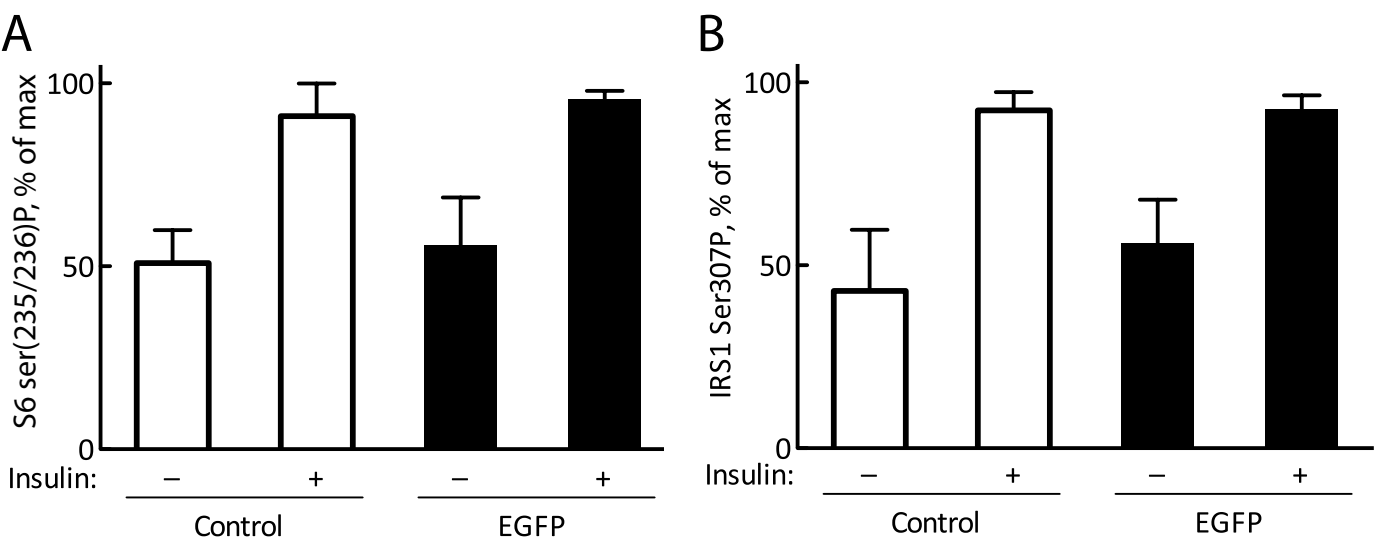

Supplement: Figure S1 — Overexpression of adenovirus expressing EGFP does not affect S6 or IRS1 Ser307 phosphorylation. Isolated human primary adipocytes were infected with EGFP adenovirus. Untransfected cells were used as control. Cells were stimulated with 10 nM insulin for 40 min (A) or 10 min (B). Whole cell lysates were subjected to SDS-PAGE and immunoblotted against S6 phospho-ser235/236 (A) and IRS1 phospho-ser307 (B). Mean±SE (n = 3). (PDF) [file pone.0059725.s001.pdf]
